# Revisiting the Factors Underlying Maxillary Midline Diastema

**DOI:** 10.1155/2016/5607594

**Published:** 2016-04-13

**Authors:** Abdullah M. Zakria Jaija, Amr Ragab El-Beialy, Yehya A. Mostafa

**Affiliations:** ^1^Private Practice, P.O. Box 376385, Riyadh 11335, Saudi Arabia; ^2^Department of Orthodontics and Dentofacial Orthopedics, Faculty of Oral and Dental Medicine, Cairo University, 11 El-Saraya Street, Manial, Cairo 12511, Egypt; ^3^MOrth Programs at Cairo University and Future University, Cairo, Egypt

## Abstract

*Aim*. The aim of this study is to analyze the etiological factors underlying the presence of maxillary midline diastema in a sample of orthodontic patients.* Materials and Methods*. One hundred patients who fulfill the inclusion criteria were selected from 1355 patients seeking orthodontic treatment. The pretreatment orthodontic records were analyzed. The width of the maxillary midline diastema was measured clinically with a digital caliper at two levels: the mesioincisal angles of the central incisors and five millimeters from the incisal edge. The two measurements were averaged, and patients with diastema of more than 0.5 millimeter in width were enrolled.* Results*. Diastema is a multifactorial clinical finding with more than one underlying etiological cause. The interrelationship between the familial pattern of midline diastema and the microdontia, macroglossia, labial frenum, and alveolar cleft conforms was clear. The effect of a mesiodens and the upper lateral incisor whether bilaterally missing, unerupted, or peg shaped was minimal.* Conclusion*. Etiological factors underlying maxillary midline diastema are interconnected. Using a checklist as a guide during handling maxillary midline diastema is important in the different stages of treatment.

## 1. Introduction

The presence of a midline diastema represents an esthetic and psychological impairment and distress for patients seeking orthodontic treatment [1]. Maxillary midline diastema is a clinical sign, which has a multitude of underlying etiological factors that might be interdependent or independent.

Many etiological factors for maxillary midline diastema have been reported in the literature [2]. Among them are the physiological (developmental) self-limiting diastema, familial background, mesiodens [3–5], abnormal labial frenum [6–8], missing or undersized lateral incisor, thumb sucking, mouth breathing, tongue thrust, ankylosed central incisor, flared or rotated central incisors, anodontia, macroglossia [9, 10], dentoalveolar disproportion, generalized spacing [11], localized spacing, closed bite, facial type, ethnic and familial characteristics [12, 13], interpremaxillary suture and transseptal fibers [14], midline pathology, midline submucosal alveolar cleft [15], tongue piercing [16], gingival recession, and pathological tooth migration [17].

The aim of this survey is to investigate the correlation between the different etiological factors underlying maxillary midline diastema and highlight their clinical implications.

## 2. Materials and Methods

**Figure 1:**
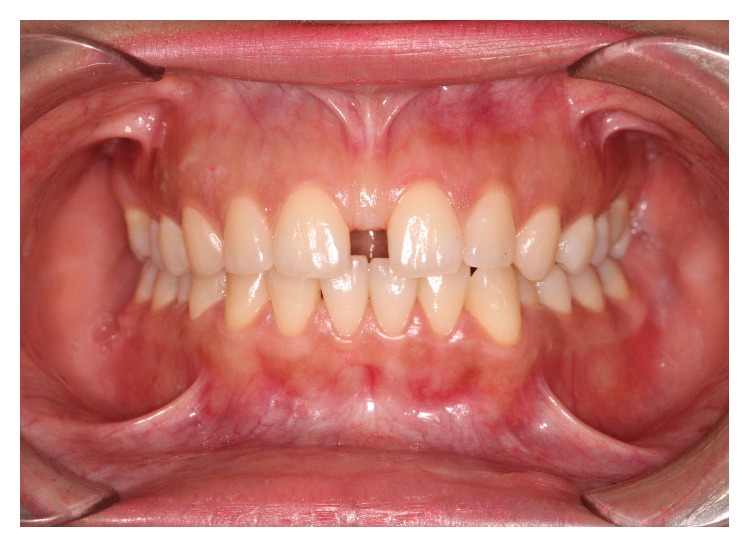
Maxillary midline diastema.

This survey was performed on patients undergoing orthodontic treatment at three orthodontic graduate programs. The pretreatment records of 1355 patients were examined, to collect 100 patients fulfilling the inclusion criterion (presence of maxillary midline diastema >0.5 mm) within an age range of 13–30 years. The pretreatment records included history, intra- and extraoral examination, and panoramic and periapical radiographs of the maxillary incisor region. The width of the maxillary midline diastema was measured clinically with a digital caliper at two levels: the mesioincisal angles of the central incisors and five millimeters from the incisal edge. The two measurements were averaged, and patients with diastema of more than 0.5 millimeter in width were enrolled (Figure 1). The examination was made by the principle observer and repeated by the second observer.

Because of the physiological diastema, patients younger than 13 years were excluded, while patients above 30 years were excluded because of the possibility of diastema formation due to periodontal involvement and migration of teeth.

**Figure 2:**
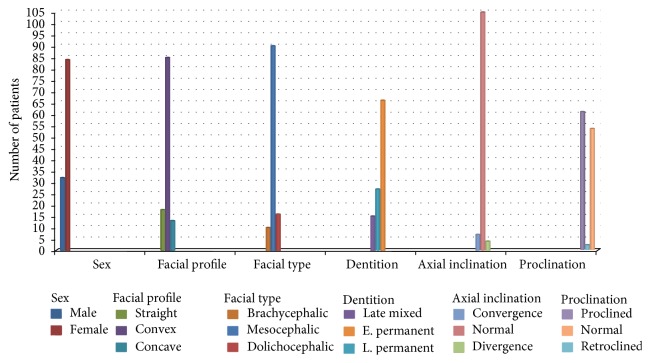
Bar chart showing the distribution of the criteria of the sample.

The distribution of the criteria of the sample was analyzed (Figure 2). The criteria represent the commonsensical orthodontic categories that segregate the sample into comparable subclasses. The etiological factors underlying the maxillary midline diastema were extracted from the records and clinical examination of the patients (Table 1). These etiological foundations were separated into major etiological factors and etiological factors of lesser influence. These factors represent all the etiological factors underlying the presence of the maxillary midline diastema that were extracted from the research sample. The prevalence of each factor in percentage of the 100 cases enrolled was calculated.

**Figure 3:**
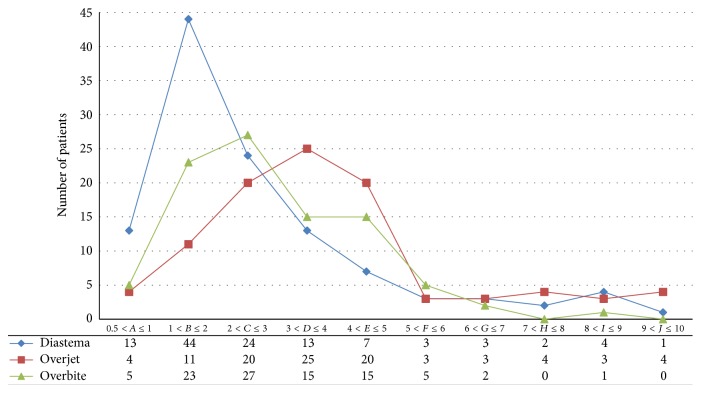
Chart showing the relation of common prevalence between diastema, overjet, and overbite.

Additionally, the association between diastema with overjet and overbite is depicted through dividing the sample into 10 groups each representing 1 mm regarding diastema, overjet, and overbite (Figure 3).

## 3. Results

The prevalence of the diastema was found to be 13.6% among the screened sample. The occurrence of the six criteria (Figure 2) demonstrated that the maxillary midline diastema is more observed in females, mesocephalic faces, convex facial profiles, and the early permanent dentition. Maxillary midline diastema is more prevalent with upright maxillary central incisors than convergent or divergent central incisors. The least prevalence of diastema occurs with retroclined maxillary incisors.

The relationship between the maxillary midline diastema, overjet, and overbite depicted in Figure 3 shows that a diastema width of 1-2 mm is more prevalent (44 patients) than other extents of diastema, and this prevalence decreases as the amount of overbite and overjet increases.

**Figure 4:**
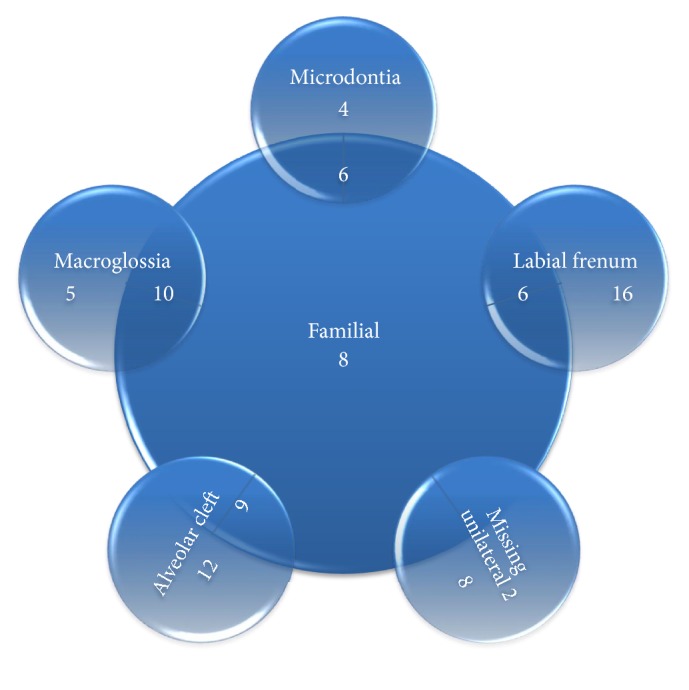
Intersecting circles chart depicting the common occurrence between the major contributing factors.

**Figure 5 fig5:**
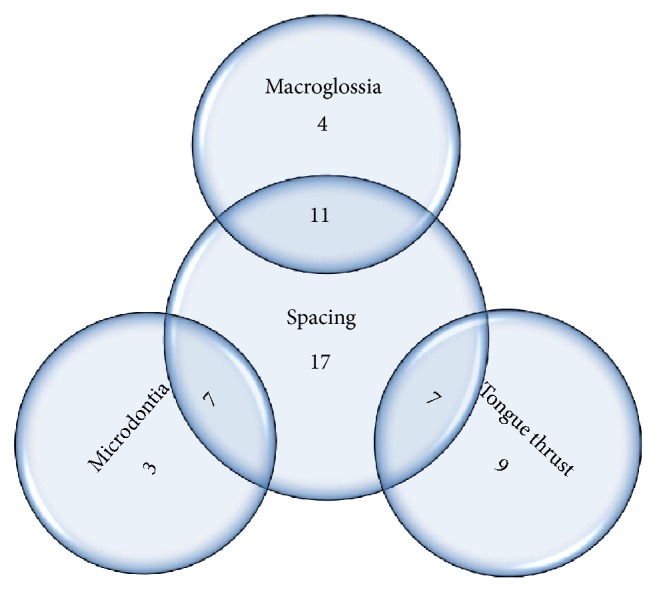
Intersecting circles chart depicting the common occurrence between spacing, microdontia, macroglossia, and tongue thrust.

**Figure 6:**
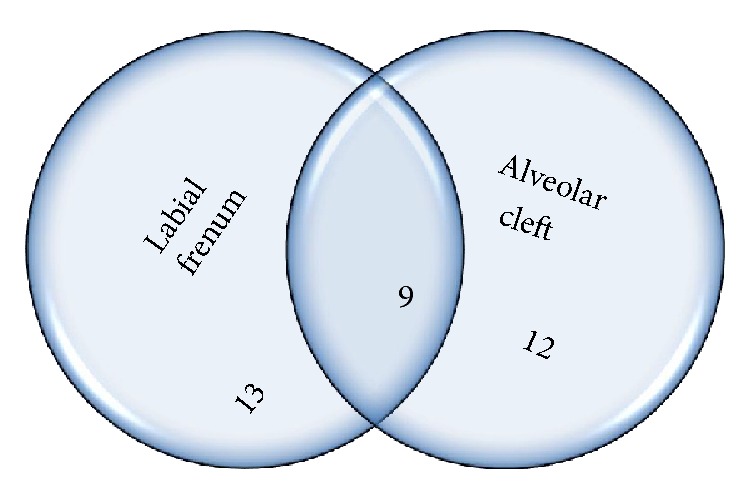
Intersecting circles chart depicting the common occurrence between labial frenum and alveolar cleft.

Etiological factors were segregated into major contributing factors and factors of lesser contribution taking 5% prevalence as the limit (Table 1). The interrelation (overlap) between the major contributing factors is denoted by intersecting circles charts (Figures 4 [Fig fig5]–6). Factors that might be of strong developmental interrelation were linked together in a single chart. The areas of intersection represent the number of cases where more than one major contributing factor exists.

## 4. Discussion

Treatment of maxillary midline diastema should be directed towards management of the underlying cause before seeking closure of the diastema; thus, identifying the etiology is of chief importance. The aim of this survey is to highlight the factors underlying maxillary midline diastema and the interrelation between them. This might influence the timing for closure of the diastema during treatment and/or retention protocols.

Our results conformed to the consensus that diastema is a multifactorial clinical finding with more than one underlying etiological cause. Based on a prevalence of 5%, the etiological factors were segregated into major and minor factors. Surprisingly, the effect of the upper lateral incisor whether bilaterally missing, unerupted, or peg shaped was minimal. The same outcome was found with a mesiodens. The interrelationship between the familial pattern of midline diastema and the microdontia, macroglossia, labial frenum, and alveolar cleft conforms was clear. On the other hand, no cases showed a familial tendency of missing unilateral maxillary lateral incisor. However, as regards the enlarged labial frenum as an etiological cause, results of this study revealed that it represents only a minor etiological cause, an observation that conforms to the findings of Huang and Creath [2]. In addition, the interrelation between the alveolar cleft and abnormal labial frenum was an important finding.

Implementation of the findings of this survey is important from the clinical sense. The impact of each etiological factor of the maxillary midline diastema upon the diagnosis, treatment, or retention protocol is summarized into a checklist. This checklist was designed to highlight the intervention at the different stages of treatment for each etiological factor (Table 2).

## 5. Conclusion

Etiological factors underlying maxillary midline diastema are interconnected.

Using a checklist as a guide during handling maxillary midline diastema is important in the different stages of treatment.

## Figures and Tables

**Table 1:** The etiological factors of the maxillary midline diastema extracted from the sample.

Major etiological factors	% of prevalence	Etiological factors of lesser prevalence	% of prevalence
Generalized spacing	42	Missing bilateral maxillary central incisors	5
Familial incidence	39	Bilateral missing maxillary lateral incisors	5
Abnormal frenal attachment	22	Peg shaped maxillary lateral incisors	5
Alveolar intraosseous cleft	21	Missing unilateral maxillary central incisors	4
Tongue-thrusting	16	Ankylosed maxillary central incisors	1
Macroglossia	15	Excess bony defect	1
Unerupted canine bilateral	14	Thumb sucking	1
Unerupted canine (unilateral)	12	Mesiodens	1
Microdontia	10	Malformed maxillary central incisors	0
Unilateral missing maxillary lateral incisors	8	Genetics	0
Palatally erupted maxillary lateral incisors	6	Midline pathosis	0
Mouth breathing	6	Unerupted maxillary lateral incisors	0
Tooth migration	6		

**Table 2:** Checklist showing the impact of each etiological factor of the maxillary midline diastema upon the diagnosis, treatment, or retention protocol.

Factor	Extra diagnostic tool	Treatment modification	Retention modification
Generalized spacing			⬜ (Permanent)
Familial incidence	⬜ (Family screening)		⬜ (Permanent)
Abnormal frenal attachment	⬜ (Periapical radiograph)	⬜ (Frenotomy)	⬜ (Prolonged)
Alveolar intraosseous cleft	⬜ (Periapical radiograph)	⬜ (Nonidentified)	
Tongue-thrusting		⬜ (Habit breaking appliance)	⬜ (Habit breaking)
Macroglossia		⬜ (No encroaching on tongue)	⬜ (Permanent)
Unerupted canine bilaterally			
Unerupted canine unilaterally			
Microdontia		⬜ (Build-up)	
Unilateral missing maxillary lateral incisors		⬜ (Prosthesis) ⬜ (Canine substitution)	
Palatally erupted maxillary lateral incisors		⬜ (Root torquing)	
Mouth breathing	⬜ (ENT consultation)		⬜ (Habit breaking)
Tooth migration			
Missing bilateral maxillary central incisors		⬜ (Prosthesis)	
Bilateral missing maxillary lateral incisors		⬜ (Prosthesis) ⬜ (Canine substitution)	
Peg shape maxillary lateral incisors		⬜ (Build-up)	
Missing unilateral maxillary central incisors		⬜ (Prosthesis)	
Ankylosed maxillary central incisors		⬜ (Luxation, crowning, extraction)	
Excess bony defect		⬜ (Surgical)	
Thumb sucking		⬜ (Habit breaking appliance)	
Mesiodens		⬜ (Surgical extraction)	
Malformed maxillary central incisors		⬜ (Build-up)	
Midline pathosis	⬜ (Periapical radiograph)	⬜ (Surgical excision)	
Unerupted maxillary lateral incisors	⬜ (Periapical radiograph)		
